# Predictors of Long-Term Improvement Following Cognitive Remediation in a Sample With Elevated Depressive Symptoms

**DOI:** 10.3389/fpsyg.2020.02232

**Published:** 2020-09-11

**Authors:** Bjørn Ingulfsvann Hagen, Nils Inge Landrø, Bjørn Lau, Ernst H. W. Koster, Jan Stubberud

**Affiliations:** ^1^Department of Research, Lovisenberg Diaconal Hospital, Oslo, Norway; ^2^Department of Psychology, University of Oslo, Oslo, Norway; ^3^Department of Experimental Clinical and Health Psychology, Ghent University, Ghent, Belgium

**Keywords:** depression, cognitive remediation, treatment predictors, executive functions, attention

## Abstract

**Objective:**

Cognitive remediation (CR) techniques (interventions to enhance cognitive functioning) have proven moderately effective in improving cognition and daily functioning in major depressive disorder (MDD). However, baseline predictors of treatment response are lacking. The present study aimed to identify factors influencing long-term CR outcomes in a sample with current or previous, mild or moderate MDD and with self-reported cognitive deficits.

**Methods:**

Forty-two completers of group-based CR (strategy learning or drill-and-practice), were pooled into one sample. Based on change scores from baseline to 6-month follow-up, participants were categorized as “improvers” or “non-improvers” using reliable change index calculations. Measures included a questionnaire of everyday executive functioning and a neuropsychological test of attention. Finally, improvers and non-improvers were compared in terms of various sociodemographic, psychological, illness-related, and neuropsychological baseline variables.

**Results:**

Seventeen participants improved reliably in everyday executive functioning, and fourteen demonstrated a reliable improvement in attention. No statistically significant differences emerged between improvers and non-improvers.

**Conclusion:**

No major predictors of CR were identified. Importantly, the current findings are insufficient to guide clinical decision-making. Large-scale studies with *a priori* hypotheses are needed to make advances in the future.

## Introduction

Major depressive disorder (MDD) is characterized by deficits in cognitive functions, including attention, memory, and executive functions (EF) (Snyder, 2012; Ahern and Semkovska, 2017). However, the heterogeneity of the cognitive profile in MDD appears to be large, with distinct neurocognitive subgroups (Pu et al., 2018). For those experiencing cognitive difficulties, deficits often persist into remission (Rock et al., 2014) and have a deleterious effect on everyday functioning (Baune et al., 2010). Cognitive deficits, particularly in EF, are additionally associated with unfavorable depression outcomes, such as impaired long-term recovery (Vicent-Gil et al., 2018) and reduced antidepressant medication effectiveness (Groves et al., 2018). Hence, cognitive functioning represents a potential treatment target in MDD (Kaser et al., 2017).

Nonetheless, both antidepressant medication and cognitive–behavioral therapy (CBT) show limited effectiveness in alleviating cognitive deficits (Porter et al., 2016; Shilyansky et al., 2016). Cognitive remediation (CR) interventions – specifically targeting cognitive dysfunction – intended to produce lasting improvements in everyday functioning are thus emerging. Although the heterogeneity of treatment approaches labeled “CR” is large (Motter et al., 2016), these interventions can be divided into either bottom-up drill-and-practice approaches or top-down approaches focusing on strategy learning. Bottom-up approaches typically consist of computerized cognitive training (CCT) tasks intended to improve basic cognitive processes through the process of neuroplasticity (Motter et al., 2016). In contrast, top-down approaches consist of learning compensatory strategies for wide appliance in daily living, to compensate for the cognitive difficulties. Findings indicate moderate effectiveness in improving cognition and everyday functioning following CR, but there is a paucity of evidence for improved EF or on its long-term effects (for a meta-analysis, see Motter et al., 2016). In this context, it has been argued that the substantial heterogeneity in the cognitive profile of MDD may influence the effectiveness of CR (Koster et al., 2017).

The lack of factors associated with successful treatment outcomes may be a barrier to improving CR effectiveness (Motter et al., 2016; Koster et al., 2017). The identification of pretreatment predictors could improve efficacy by facilitating individualized clinical decision-making. Moreover, it may be helpful for the development of future treatments by providing insight into the mechanisms of CR interventions (Koster et al., 2017). The investigation of CR predictors and moderators in MDD is scarce, but a meta-analysis covering a range of CCT interventions found decreased treatment effectiveness with increased age (Motter et al., 2016). Additional variables, such as gender and receiving concurrent treatment (antidepressant medication or psychotherapy), did not significantly influence outcomes. In a recent study dedicated to identifying the predictors in a CR intervention consisting of both CCT and strategy learning, Listunova et al. (2020) observed a shorter duration of illness to be the only factor associated with improvement on a neuropsychological measure of attention in a partially remitted MDD sample. Several sociodemographic, neurocognitive, psychopathological, and training-specific factors thus failed to predict outcomes. However, the study was limited by its exploratory approach, modest sample size, and exclusive focus on the attention domain (Listunova et al., 2020). Interestingly, CR findings diverge from psychotherapy research in MDD, where illness characteristics such as greater depression symptom severity, younger age at onset, and more previous episodes have all been associated with poorer responses in relation to depressive symptom alleviation (Hamilton and Dobson, 2002). Moreover, in schizophrenia research, where predictors and moderators of CR effectiveness have been more frequently studied, most of the factors reviewed fail to significantly influence CR treatment response (Reser et al., 2019; Seccomandi et al., 2020). However, in a selection of studies, better baseline performance in several cognitive domains predicted both cognitive and functional improvement, while increased chronicity and severity of schizophrenia has been associated with worse CR outcomes (Medalia and Richardson, 2005; Kurtz et al., 2009; Vita et al., 2013; Lindenmayer et al., 2017).

The main aim of the present study was to explore whether a selection of sociodemographic, neuropsychological, illness-related, and psychological variables could predict long-term CR outcomes in a sample with current or previous mild or moderate MDD. Data were collected as part of a single-blind randomized controlled trial (RCT) comparing the effectiveness of a strategy-based CR approach, Goal Management Training (GMT), with drill-and-practice CCT, in improving EF (Hagen et al., 2020). Both groups improved on measures of EF in daily life, neuropsychological tests of EF and attention, and depression symptom severity following CR. That is, no significant differences emerged between groups in the original study, although within-group changes in everyday EF and depression symptom severity were only significant following GMT. Owing to the limited number of previous studies examining predictors of CR in MDD, the present study applied an exploratory approach with no *a priori* hypothesis.

## Materials and Methods

The original RCT was preregistered at clinicaltrials.gov with the identifier NCT03338413, and the study protocol was approved by the Regional Committee for Medical and Health Research Ethics, South-Eastern Norway (2017/666). The study was conducted following the World Medical Association’s Declaration of Helsinki, and all participants gave their written informed consent. For more detailed information on the methodological approach of the original RCT study, see Hagen et al. (2020).

### Participants

The sample (*n* = 63) included participants diagnosed with mild or moderate MDD according to *International Classification of Diseases* – *10th Revision* (*ICD-10*) criteria (World Health Organization, 2004), either as a primary or as a secondary diagnosis. All participants had undergone a diagnostic evaluation and completed treatment at the Return-to-Work clinic at Lovisenberg Diaconal Hospital within 2 years before inclusion. The Return-to-Work clinic offers short-term outpatient psychotherapeutic treatment to patients with mental health issues of mild to moderate severity, at risk of receiving sick leave because of mental illness. In addition, to be included, participants had to be between 18 and 60 years of age and to have self-reported everyday EF deficits (e.g., difficulties with memory, organizing/planning, emotional regulation, and/or concentration) in a custom-made telephone interview. All participants were additionally asked to confirm that depression symptoms represent a major mental health complaint. Exclusion criteria included comorbid neurological conditions, ongoing alcohol or substance abuse, and severe cognitive problems or mental disorders (psychotic disorders and severe personality disorders). No cutoff scores were specified for current depressive symptom severity. There were no restrictions on participants concerning additional concurrent psychotherapeutic or antidepressant treatment.

### Study Design and Blinding

All participants completed a baseline assessment (T1) before being randomly assigned to nine sessions of either GMT or CCT, using computer-generated simple randomization. Participants were reassessed immediately following treatment completion (T2) and at a 6-month follow-up (T3). The assessments consisted of neuropsychological tests and self-report rating scales. Assessments were not blind, because the person responsible for data collection also acted as a therapist in both interventions. To compensate, an external assessor performed a limited set of blind T3 assessments (*n* = 5) for comparison. However, the study was single-blind because participants were not informed whether they had been allocated to the condition considered to be the active treatment.

### Cognitive Remediation Interventions

#### Goal Management Training

GMT is a manual-based CR intervention to improve everyday EF (Levine et al., 2011). A central element of GMT is to learn and internalize strategies for wide application in daily living, promoting goal-directed behavior through increased executive control and improved problem-solving capacities. Strategies consist of a self-instruction to stop ongoing behavior, check the current content of working memory, state and define goals, apply a systematic approach to problem-solving, and monitor performance.

In the present study, the Norwegian translation of the standard GMT protocol was employed (Stubberud et al., 2013; Tornås et al., 2016). A clinical psychologist and a neuropsychologist delivered GMT in groups of five to seven participants in 9 weekly 2 h sessions. In-class exercises included practicing the use of the compensatory strategies (e.g., practice a systematic approach to problem-solving by arranging a fictitious wedding party). Mindfulness exercises (Kabat-Zinn, 1990), intended to enhance attentional control, were also practiced in class. Sessions emphasized group discussions addressing personal examples of dysexecutive behavior. Between-session assignments included monitoring EF-related errors, mindfulness exercises, and the application of learned strategies in daily life (Table 1).

**TABLE 1:** GMT sessions, CCT tasks, and objectives.

GMT session	Objective	CCT task	Objective
The present and the absent mind	Tracking absentmindedness/Practice mindfulness techniques	Double decision	Attention
Inattentive Errors	Condition for, and consequences of, absentmindedness	Divided attention	Attention/Inhibition
The Automatic Pilot	How automatic behavior leads to inappropriate responding	Target tracker	Attention
Stop the automatic pilot	Make a habit of stopping/Bring attention to the present	Syllable stacks	Working memory
The mental blackboard	Check the content of working memory	Scene crasher	Working memory
State your goal	State goals to facilitate goal maintenance	Face-to-Face	Processing Speed/Social Cognition
Making decisions	Goal conflict in decision-making/Making to-do lists	Sound sweeps	Processing speed
Splitting tasks into subtasks	Split overwhelming tasks into subtasks/Step-by-step approach		
Checking (STOP!)	Adapting behavior to situational change/Summary of training		

*GMT, Goal Management Training; CCT, computerized cognitive training.*

#### Computerized Cognitive Training

The CCT consisted of seven exercises from the BrainHQ platform. Repetition is the hallmark feature of CCT, and neuroplasticity is its theoretical foundation (Siegle et al., 2007). Cognitive improvements, including EF, have been identified using BrainHQ or similar exercises (Morimoto et al., 2014; Lewandowski et al., 2017), and these studies established the empirical basis for the selection of exercises. In addition, exercise selection was based on the provider’s description^1^.

The CCT consisted of nine twice-weekly 1 h sessions by groups of three participants. Exercises targeted attention, memory, processing speed, and EF. To ensure appropriate levels of mastery and frustration, the platform adapted difficulty levels to the individual participants’ performance, keeping the success rate at 80% throughout. A clinical psychologist acted as a therapist and gave participants positive feedback on their efforts. The first session included psychoeducation, with the therapist introducing the concept of neuroplasticity, typical cognitive deficits in depression, and the importance of cognitive processes in different everyday situations. Participants had online access to the training platform and were encouraged to practice for at least 30 min between each session (Table 1).

### Completer Sample

**FIGURE 1:**
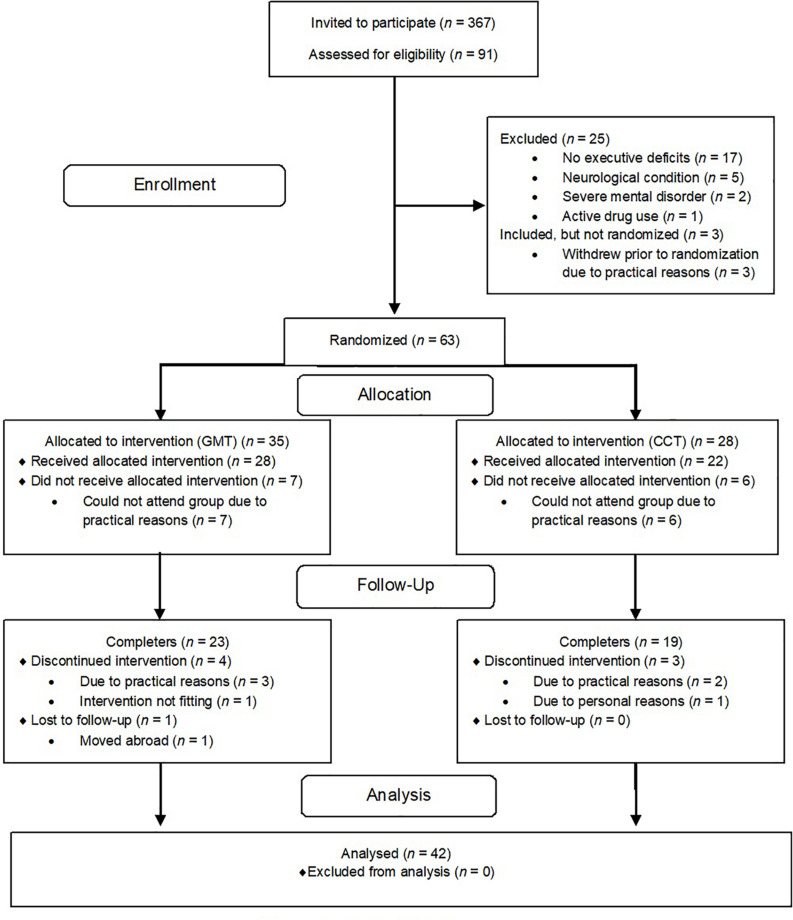
CONSORT diagram (Schulz et al., 2010).

Participants had to attend a minimum of six training sessions and complete the 6-month follow-up assessment (T3) to be included in the completer sample. Forty-two completers from both groups were pooled into one sample. The pooling of participants receiving different treatments was done to increase the sample size (Figure 1).

### Outcome Measures

The Global Executive Composite (GEC) from the Behavior Rating Inventory of Executive Function – Adult version (BRIEF-A) (Roth et al., 2005) was applied as an outcome of self-reported everyday EF. The BRIEF-A GEC consists of 70 items and nine non-overlapping subscales (Inhibit, Self-Monitor, Plan/Organize, Shift, Initiate, Task Monitor, Emotional Control, Working Memory, Organization of Materials), tapping the frequency of everyday dysexecutive behavior (item range 1–3, total range 70–210). The psychometric properties of the GEC are acceptable, with a 1-month test–retest reliability of 0.94 and a Cronbach’s alpha of 0.96 (Roth et al., 2005).

The Conners Continuous Performance Test – Third edition (CPT-3) (Conners, 2015) was applied as a neuropsychological measure of attention. The CPT-3 is a 14 min go/no-go test of visual attentiveness, response inhibition, and sustained attention. The number of commission errors (Commissions; response to “no-go” targets: attentiveness and inhibition) and hit reaction time standard deviation (HRT SD: response consistency/sustained attention) subscales were included as outcome measures. The corrected test–retest reliabilities (1–5 weeks) of the included subscales are 0.68 (HRT SD) and 0.85 (Commissions) (Conners, 2015). A neuropsychological measure of attention was selected as an outcome to facilitate comparison with the only previous study that we know of that investigated the predictors of CR response in MDD (Listunova et al., 2020).

### Predictors of Treatment Effect

#### Sociodemographic Factors

Sociodemographic factors included age, gender, years of education, and employment status, all self-reported in a custom-made interview. Employment status was transformed into a dichotomous variable with the categories “full-time employment/full-time student” and “other” (including “part-time employment/part-time student,” “sick leave,” and “looking for a job”).

#### Illness-Related Factors

Illness-related factors included current depressive symptom severity in addition to the self-reported number of previous depressive episodes, age of onset, duration of illness, and current antidepressant medication use. Depressive symptom severity was assessed with the Beck Depression Inventory (BDI) (Beck et al., 1961), which has satisfactory internal consistency (Beck et al., 1988). The remaining illness-related factors were self-reported during a custom-made interview. The number of previous episodes was transformed into a dichotomous variable based on the categories of “one episode” and “more than one episode,” as this was considered a theoretically meaningful subdivision of the highly skewed original continuous variable.

#### Psychological Factors

Psychological factors included overall psychological distress and a tendency to ruminate. The Clinical Outcomes in Routine Evaluation – Outcome Measure (CORE-OM) (Barkham et al., 2001) was applied as a measure of overall psychological distress. The CORE-OM clinical score was calculated as a mean of completed items multiplied by 10 (range: 0–40). Rumination was assessed using the Ruminative Response Scale (RRS) (Treynor et al., 2003). Both questionnaires have acceptable internal consistency (Barkham et al., 2001; Treynor et al., 2003).

#### Neuropsychological Factors

Neuropsychological factors included estimated IQ, assessed with the two-subtest form (Matrix reasoning; Vocabulary) of the Wechsler Abbreviated Scale of Intelligence (WASI) (Wechsler, 1999). Performance on the Delis–Kaplan Executive Function System (D-KEFS) (Delis et al., 2001) Trail-Making Test was applied as a measure of processing speed (condition 2) and EF/shifting (condition 4). Memory was assessed using the California Verbal Learning Test–Second edition – Short form (CVLT-II SF) (Delis et al., 2000). The digit span forward (attention span) and digit span backward (working memory) subtests of the Wechsler Adult Intelligence Scale – Fourth edition (WAIS-IV) (Wechsler, 2014), were also employed.

#### Other Factors

Received intervention (GMT or CCT) was included as a training-specific factor. In addition, baseline performance scores on the outcome variables were included as predictors.

### Statistical Analysis

#### Calculation of Reliable Change Index

The reliable change index (RCI) (Jacobson and Truax, 1991) was calculated for everyday EF (BRIEF-A GEC) and the neuropsychological measure of attention (CPT-3: Commissions, HRT SD). The RCI analysis is a statistical approach to identify individuals with statistically reliable improvement, given the scale reliability. Thus, the approach is sensitive to individual participant improvements potentially lost in group-level statistical analysis (Jacobson and Truax, 1991). To calculate the RCI, a change in individual raw score (BRIEF-A) or *T*-score (CPT-3) between T1 (*X*_1_) and T3 (*X*_2_) was divided by the standard error of the difference (SE_diff_) using the formula:

RCI=(X-2X)1/SEd⁢i⁢f⁢f

*SE*_diff_ was derived from the standard error of measurement (*S*_E_), calculated using the test–retest reliability (*r*_xx_) of the instrument, and the standard deviation (*SD*) using the following formulas:

S=ES(1-rx⁢x)andSE=d⁢i⁢f⁢f2⁢(SE)2

An RCI smaller than –1.96 (because of measurement direction) was required to be considered as a reliable improvement. A change surpassing the ±1.96 threshold occurs by chance in only 5% of cases. Information on test–retest reliability and standard deviation was collected from the test manuals (Roth et al., 2005; Conners, 2015). The decision to calculate change scores from baseline (T1) to the 6-month follow-up (T3) was because long-term outcomes were regarded as most clinically relevant. For the CPT-3, a reliable improvement on one of the two subscales (Commissions or HRT SD) was required to count as an improvement. Finally, participants improving reliably on one subscale were not included if there was a reliable deterioration on the other.

#### Comparison of Improvers and Non-improvers

For each of the two outcome measures, participants were categorized as either “improvers” or “non-improvers” based on their RCI score. Improvers were compared with non-improvers in the pooled completer samples using the non-parametric Mann–Whitney *U* test and chi-square test, for pairs of continuous and dichotomous variables, respectively. In addition, the T3 results on the outcome measures were compared between assessors (blind/non-blind), and T1 results between completers and non-completers, using the Mann–Whitney *U* test. All tests were two-tailed, and to partially account for multiple testing, the significance level was set to 0.01. Values between 0.01 and 0.05 were interpreted as trends. SPSS version 24.0 for Windows was applied for all analyses.

## Results

The completers (*n* = 42) had a median age of 41 years (range = 28–59) and a median of 15 years of education (range = 9–18). The majority were female (79.1%), and 76.7% did not currently use antidepressant medication. Furthermore, their average depression symptom severity was in the mild range (BDI: median = 17.0, range = 4.0–34.0) (Beck et al., 1988). Fourteen completers had been diagnosed with a comorbid *ICD-10* mental or behavioral disorder (Table 2).

**TABLE 2:** Comorbid *ICD-10* diagnoses in the completer sample.

Disorder	Frequency
Generalized anxiety disorder (F 41.1)	6
Panic disorder (F 41.0)	3
Post-traumatic stress disorder (F 43.1)	1
Agoraphobia (F 40.0)	1
Social phobia (F 40.1)	1
Anxiety disorder, unspecified (F 41.9)	1
Nonorganic insomnia (F 51.0)	2
Mental and behavioral disorders due to psychoactive substance use (F 10–F 19)	2

*Number of participants with comorbidities, n = 14. Multiple comorbid diagnoses are possible for each participant. ICD-10 = International Classification of Diseases – 10th Revision.*

The sample reported substantial executive dysfunction in daily living (BRIEF-A GEC *T*-score: median = 64, range = 44–80) but performed in the normal range for the included CPT-3 subscales at baseline (Commissions *T*-score: median = 48, range = 35–73; HRT SD *T*-score: median = 44, range = 33–75). At follow-up, completers reported overall fewer EF deficits in daily life (BRIEF-A GEC *T*-score: median = 59.5, range = 36–78) and performed better on the measure of attention (CPT-3: Commissions *T*-score, median = 44, range = 25–71; HRT SD *T*-score, median = 41, range = 31–56). No statistically significant differences emerged for any of the outcome measures at follow-up between the blinded and non-blinded assessors. Finally, the completers were not significantly different from the non-completers (*n* = 21) on any of the included variables.

### Comparison of Everyday Executive Functioning of Improvers and Non-improvers

Seventeen participants (40.5%) were identified to improve reliably on the BRIEF-A GEC between T1 and T3. For participants to surpass the critical value for improvement, a 13-point reduction in BRIEF-A GEC raw score was required. The mean BRIEF-A GEC raw score of improvers at follow-up was 107.9 (*SD* = 17.9). No statistically significant differences emerged between improvers and non-improvers for any of the predictors. However, improvers had a higher estimated IQ than non-improvers at trend level (*p* = 0.044) (Table 3).

**TABLE 3:** Characteristics of improvers and non-improvers in everyday EF.

Variable	Improvers	Non-improvers	*p*-value
	(*n* = 17)	(*n* = 25)	
**Categorical variables**			
Gender (female)	14	19	0.622
Work status (full-time)	11	18	0.616
Antidepressant use (yes)	2	8	0.131
Dep. ep. (1 ep.)	5	9	0.754
Received intervention (GMT)	10	13	0.663

**Continuous variables**	**Mean (*SD*)**	**Mean (*SD*)**	

Age (years)	44.9 (8.7)	40.8 (8.5)	0.098
Years of education	15.5 (2.0)	14.4 (2.1)	0.071
Age of onset (years)	32.7 (12.9)	26.0 (10.3)	0.087
Illness duration (years)	12.2 (10.4)	14.8 (9.7)	0.317
WASI (IQ estimate)	115.3 (6.7)	108.8 (12.1)	0.044
TMT 2 (seconds)	30.8 (10.2)	31.4 (9.7)	0.488
TMT 4 (seconds)	71.2 (21.6)	84.1 (34.6)	0.109
CVLT-II SF Long delay	8.4 (1.2)	8.3 (0.8)	0.299
WAIS-IV DS Forward	9.7 (2.4)	9.1 (1.5)	0.539
WAIS-IV DS Backward	10.0 (2.3)	9.8 (1.8)	0.815
BDI	19.0 (6.7)	15.2 (7.1)	0.063
RRS	56.3 (10.5)	55.0 (12.2)	0.710
CORE-OM	14.7 (5.5)	13.4 (6.0)	0.522
CPT-3 Commissions (*T*-score)	50.2 (11.3)	51.5 (8.4)	0.336
CPT-3 HRT SD (*T*-score)	46.6 (9.8)	44.8 (9.8)	0.293
BRIEF-A GEC	137.3 (17.9)	128.6 (15.2)	0.124

*SD, standard deviation, Dep., depression; ep., episode; GMT, Goal Management Training; WASI, Wechsler Abbreviated Scale of Intelligence; TMT, Trail-Making Test; CVLT-II SF, California Verbal Learning Test–Second edition – Short form; WAIS-IV, Wechsler Adult Intelligence Scale – Fourth edition; DS, digit span; BDI, Beck Depression Inventory; RRS, Ruminative Response Scale; CORE-OM, Clinical Outcomes in Routine Evaluation – Outcome Measure; CPT, Conners Continuous Performance Test; HRT, hit reaction time; BRIEF-A GEC, Behavior Rating Inventory of Executive Function – Adult version Global Executive Composite; EF, executive functions. All scores reported are raw scores, except when otherwise specified. Higher scores indicate greater impairment, except for WASI, CVLT-II, and WAIS-IV.*

### Comparison of Attention by Improvers and Non-improvers

Fourteen participants (33.3%) were identified as improvers on the measure of attention. Twelve participants improved on the Commissions subscale and four on the HRT SD subscale, while two participants improved on both subscales. For participants to surpass the critical value for improvement, a change in *T*-score of 10 (Commissions) or 13 (HRT SD) was required. At follow-up, the mean *T*-score for the improver group (*n* = 14) was 42.2 (*SD* = 6.5) on the Commission subscale and 39.9 (*SD* = 7.5) on the HRT SD subscale. No statistically significant differences emerged between improvers and non-improvers. However, at trend level, fewer of the improvers compared with the non-improvers (*p* = 0.011) had experienced only one previous depressive episode (Table 4). The mean number of self-reported previous depressive episodes was 4.8 (*SD* = 3.3) for improvers and 3.4 (*SD* = 3.2) for non-improvers (values > 10 were recoded > 10 = 10).

**TABLE 4:** Characteristics of improvers and non-improvers in attention.

Variable	Improvers	Non-improvers	*p*-value
	(*n* = 14)	(*n* = 28)	
**Categorical variables**			
Gender (female)	11	22	0.999
Work status (full-time)	10	19	0.813
Antidepressant use (yes)	1	9	0.073
Dep. ep. (1 ep.)	1	14	0.011
Received intervention (GMT)	7	16	0.661

**Continuous variables**	**Mean (*SD*)**	**Mean (*SD*)**	

Age (years)	42.9 (9.4)	42.3 (8.5)	0.915
Years of education	15.1 (2.1)	14.8 (2.1)	0.539
Age of onset (years)	29.3 (13.6)	28.4 (11.0)	0.957
Illness duration (years)	13.6 (9.3)	13.9 (10.4)	0.947
WASI (IQ-estimate)	114.9 (6.4)	109.6 (12.0)	0.194
TMT 2 (seconds)	30.3 (7.6)	31.5 (10.8)	0.904
TMT 4 (seconds)	79.1 (21.4)	79.2 (34.3)	0.566
CVLT-II SF Long delay	8.3 (0.9)	8.3 (1.0)	0.801
WAIS-IV DS Forward	10.1 (2.1)	9.0 (1.7)	0.092
WAIS-IV DS Backward	10.5 (1.8)	9.6 (2.0)	0.151
BDI	18.8 (6.5)	15.7 (7.3)	0.169
RRS	58.4 (10.7)	54.1 (11.7)	0.279
CORE-OM	16.5 (4.9)	12.6 (5.9)	0.056
CPT-3 Commissions (*T*-score)	54.8 (9.3)	49.0 (9.3)	0.050
CPT-3 HRT SD (*T*-score)	49.5 (13.3)	43.5 (6.7)	0.224
BRIEF-A GEC	137.6 (14.5)	129.4 (17.3)	0.157

*SD, standard deviation; Dep., depression; ep., episode; GMT, Goal Management Training; WASI, Wechsler Abbreviated Scale of Intelligence; TMT, Trail-Making Test; CVLT-II SF, California Verbal Learning Test–Second edition – Short form; WAIS-IV, Wechsler Adult Intelligence Scale – Fourth edition; DS, digit span; BDI, Beck Depression Inventory; RRS, Ruminative Response Scale; CORE-OM, Clinical Outcomes in Routine Evaluation – Outcome Measure; CPT, Conners’ Continuous Performance Test; HRT, hit reaction time; BRIEF-A GEC, Behavior Rating Inventory of Executive Function – Adult version Global Executive Composite. All scores reported are raw scores, except when otherwise specified. Higher scores indicate greater impairment, except for WASI, CVLT-II, and WAIS-IV.*

### Overlap Between Improvers Across Outcomes

Fifteen participants (35.7%) improved on neither measure, and four participants (9.5%) improved reliably on both the BRIEF-A GEC and the CPT-3.

## Discussion

The present study aimed to identify factors predicting long-term treatment outcomes following CR in an MDD sample. None of the variables emerged as major predictors of change in either everyday EF or attention. The lack of factors associated with CR improvement is generally consistent with previous research on MDD (Motter et al., 2016; Listunova et al., 2020).

Even though none of the illness-related factors emerged as major predictors, surprisingly, a reliable improvement in attention was associated at trend level with having experienced more than one previous depressive episode. Recurrence of episodes arguably indicates greater illness severity and chronicity, previously associated with reduced CR effectiveness for both MDD (Listunova et al., 2020) and schizophrenia (Medalia and Richardson, 2005; Vita et al., 2013; Lindenmayer et al., 2017). This result thus diverges from a selection of previous findings. However, contrary to conclusions from systematic reviews in MDD (Rock et al., 2014; Ahern and Semkovska, 2017), the present sample did not display objective attention deficits at baseline. Additionally, previous research has suggested distinct neurocognitive subgroups for MDD, with a majority showing near-normative performance on neuropsychological tests (Pu et al., 2018). Participants in the Return-to-Work program report less overall illness severity and are more likely to hold a job compared with other outpatients (Victor et al., 2016). In addition, IQ estimates were above average in the present sample. Such sample characteristics could have contributed to normal performance on the cognitive measures (Elgamal et al., 2010; Venezia et al., 2018). Furthermore, owing to the weak correlations between self-reported and neuropsychological measures of cognition in MDD, the inclusion based on subjective deficits may have also resulted in a subgroup of cognitively unimpaired participants (Petersen et al., 2019). Notably, at baseline, the non-improver group performed even better than improvers on the outcome measure of attention and may as such represent a part of the sample without actual attention deficits. The non-improvers were thus less likely to surpass the threshold for improvement, because their baseline left little room for further gains. Indeed, not accounting for individual difference in baseline performance is a limitation of the RCI approach (Duff, 2012). Moreover, although overall results are mixed, some previous findings indicate that MDD recurrence is related to impaired performance on measures of cognition (Hasselbalch et al., 2011), and this could potentially explain why recurrent episodes were associated with attention improvement in our sample when members of this group had the opportunity to improve their attention as a result of the interventions.

The present study failed to replicate the single significant finding from a previous meta-analysis investigating moderators of CR outcomes in MDD, namely, that treatment effectiveness decreases with increasing age (Motter et al., 2016). Excluding participants above the age of 60 years restricted the age range and reduced the sample variability of the present study, potentially limiting the prospect of obtaining significant results. Nonetheless, the overall available evidence does not indicate that age is a reliable predictor of CR outcomes (Reser et al., 2019; Listunova et al., 2020; Seccomandi et al., 2020). However, in accordance with previous research on MDD (Motter et al., 2016; Listunova et al., 2020), neither gender nor receiving concurrent treatments predicted improvements in cognition or everyday EF, with the latter indicating no additive effect on cognition of combining CR with antidepressant medication.

Cognitive performance at baseline did not predict outcomes for attention or EF in our sample. This finding is contrary to a selection of findings regarding schizophrenia (Reser et al., 2019) but in accordance with MDD research (Listunova et al., 2020). One notable exception was that higher IQ estimates were associated with improvement in everyday EF at the trend level. Theoretically, a greater general ability may contribute to reaching one’s potential for applying learned strategies in daily living, thereby increasing CR effectiveness (Velligan et al., 2006). Hence, the role of IQ in CR should be further investigated in future studies.

Delivering CR therapies to patients with MDD hinges on the theoretical assumption that cognitive deficits impair everyday functioning and act as risk factors for depressive symptoms. Thus, it is striking that cognitive performance at baseline (i.e., the degree of the cognitive deficits) lacks support as a moderator of CR outcome (Koster et al., 2017). No established neuropsychological profile exists for MDD (Marazziti et al., 2010), and this heterogeneity could limit the chance of detecting reliable pretreatment cognitive predictors. Furthermore, the relationship of cognitive factors with outcomes may be non-linear, exerting different influences at different levels of each variable. To illustrate, higher baseline cognitive performance may be conceptualized both as facilitating CR gains and as restricting improvement potential (Twamley et al., 2011; Vita et al., 2013).

Rumination is proposed to have a bidirectional relationship with EF (Davis and Nolen-Hoeksema, 2000; Philippot and Brutoux, 2008), and in CCT interventions specifically addressing EF processes (i.e., cognitive control training), rumination has been found to mediate depressive symptom outcomes (Quinn et al., 2014). To our knowledge, no previous study has investigated whether baseline rumination predicts CR outcomes in cognition or functioning. Our findings suggest that baseline rumination is not a major predictor of improvements in attention or everyday EF. However, important subcomponents of the rumination construct have been identified (Treynor et al., 2003) but were not presently investigated.

Pooling participants who received CR interventions that differed in content and theoretical foundation was necessary to obtain an acceptable sample size. This may have obscured the effect of predictors on each treatment. Nevertheless, the number of improvers was similar across interventions for both outcome measures in the present study; moreover, a previous meta-analysis of schizophrenia has indicated that different approaches produce similar overall effects on measures of cognition (Wykes et al., 2011), suggesting commonalities between treatments.

### Clinical Implications and Future Directions

The percentage of improvers in the present study (33.3–40.5%) and previous studies (34.2%) (Listunova et al., 2020) indicates the potential to increase CR effectiveness in MDD. The heterogeneity of cognitive deficits in depression suggests that individualized interventions may be required, and understanding why participants achieve different outcomes represents a critical hurdle to individualizing CR. However, identifying easily available major predictors of treatment outcomes has proven to be a challenge, and current findings are insufficient to guide clinical decision-making. Moreover, no consistent barriers to improvement have been identified to date, so these findings suggest that MDD patients have the potential to improve following CR, regardless of their baseline characteristics. For advances in the field, large-scale and fine-grained investigations with *a priori* hypotheses are required (Reser et al., 2019; Seccomandi et al., 2020). In addition, as has been suggested for schizophrenia, we may need to go beyond generic demographic and clinical factors to predict CR outcomes reliably (Reser et al., 2019).

The present study focused on identifying baseline predictors that could be easily disseminated into clinical practice. Hence, it did not include several factors identified as potential mediators or moderators of CR in MDD or schizophrenia, such as the number of training sessions (Buonocore et al., 2017), motivation/engagement with training (Medalia and Richardson, 2005; Siegle et al., 2014), therapist characteristics (e.g., clinical experience) (Medalia and Richardson, 2005), and patient–therapist working alliance (Huddy et al., 2012), all candidates for further investigation.

### Strengths and Limitations

The study was based on data from a single-blind RCT, applied a multimodal selection of outcome measures, and used a stringent approach to define improvement. In addition, the study attempted to extend on previous research by applying a long follow-up period as an endpoint, aiming to identify predictors of durable change following CR. However, the following limitations should be considered when interpreting the above findings. No *a priori* hypotheses were generated, and the analyses were exploratory, calling for caution in the interpretation of results. Another notable limitation was the modest sample size, reducing statistical power and increasing the risk of type II errors. Furthermore, multiple testing inflated the risk of type I errors, even if partially accounted for by lowering the significance level.

The neuropsychological measure of attention was not corrected for practice effects (Chelune et al., 1993). Although the overall practice effects on the CPT-3 are reported to be small-to-moderate across the included subscales [*T* = 2.9 for Commissions; *T* = 0.2 for HRT SD; Conners, 2015], correcting for these would still provide reliable improvement in attention that is very hard to achieve for a substantial proportion of the sample, given the conservative RCI threshold and baseline performance in the normal range. Moreover, the lack of an adequate sample for comparison (i.e., non-intervention control or comparable norm population) restricted the advantages of applying more sophisticated statistical approaches to overcome some of the above issues.

A selection of variables was transformed into dichotomous categories, which may result in a loss of information and power (MacCallum et al., 2002). Notably, this included the “number of previous episodes” variable, found to differ between groups at trend level. The follow-up assessments lacked blinding for most of the sample, increasing the risk of biased responding. However, results from a small number of blind assessments were not significantly different from those of non-blind assessments. Finally, the illness-related variables (e.g., age of onset, number of episodes) were self-reported and thus more susceptible to bias, including memory biases.

## Conclusion

In the present study, no major predictors of long-term improvement in attention or executive functioning following CR emerged. The results are consistent with previous research, which mostly failed to identify predictors of CR treatment. Importantly, the current findings are insufficient to guide clinical decision-making, and there is a need for large-scale and fine-grained investigations to extend current knowledge.

## Data Availability Statement

The datasets for this article are not publicly available because of restrictions specified in the study consent-form, and conditions for approval from the local ethics committee, concerning patient confidentiality and participant privacy. Requests to access the datasets should be directed to Jan Stubberud, jan.stubberud@psykologi.uio.no.

## Ethics Statement

The studies involving human participants were reviewed and approved by the Regional Committee for Medical and Health Research Ethics, South-Eastern Norway (2017/666). The patients/participants provided their written informed consent to participate in this study.

## Author Contributions

BH completed the data collection and data curation, analyzed the data, and wrote the manuscript draft. The study was part of a doctoral thesis by BH. JS provided supervision, conceptualized the original trial, acted as principal investigator, and contributed with revisions of the manuscript draft. BL and NL contributed to the conceptualization of the original trial and revision of the manuscript draft. EK contributed with revisions of the manuscript draft. All authors contributed to the article and approved the submitted version.

## Conflict of Interest

The authors declare that the research was conducted in the absence of any commercial or financial relationships that could be construed as a potential conflict of interest.

## Footnotes

1 https://www.brainhq.com

## References

[B1] Ahern E. Semkovska M. (2017). Cognitive functioning in the first-episode of major depressive disorder: a systematic review and meta-analysis. *Neuropsychology* 31 52–72. 10.1037/neu0000319 27732039

[B2] Barkham M. Margison F. Leach C. Lucock M. Mellor-Clark J. Evans C. (2001). Service profiling and outcomes benchmarking using the CORE-OM: toward practice-based evidence in the psychological therapies. *J. Consult. Clin. Psychol.* 69 184–196. 10.1037/0022-006x.69.2.184 11393596

[B3] Baune B. T. Miller R. McAfoose J. Johnson M. Quirk F. Mitchell D. (2010). The role of cognitive impairment in general functioning in major depression. *Psychiatry Res.* 176 183–189. 10.1016/j.psychres.2008.12.001 20138370

[B4] Beck A. T. Steer R. A. Carbin M. G. (1988). Psychometric properties of the Beck Depression Inventory: twenty-five years of evaluation. *Clin. Psychol. Rev.* 8 77–100. 10.1016/0272-7358(88)90050-5

[B5] Beck A. T. Ward C. H. Mendelson M. Mock J. Erbaugh J. (1961). An Inventory for Measuring Depression. *Arch. Gen. Psychiatry* 4 561–571. 10.1001/archpsyc.1961.01710120031004 13688369

[B6] Buonocore M. Bosia M. Bechi M. Spangaro M. Cavedoni S. Cocchi F. (2017). Is longer treatment better? A comparison study of 3 versus 6 months cognitive remediation in schizophrenia. *Neuropsychology* 31 467–473. 10.1037/neu0000347 28150964

[B7] Chelune G. J. Naugle R. I. Lüders H. Sedlak J. Awad I. A. (1993). Individual change after epilepsy surgery: practice effects and base-rate information. *Neuropsychology* 7 41–52. 10.1037/0894-4105.7.1.41

[B8] Conners C. K. (2015). *Conners’ Continuous Performance Test 3rd editionTM(Conners’ CPT 3TM), Conners’ Continuous Auditory Test of Attention (Conners’ CATATM) Manual.* Canada: Multi-Health Systems Incorporated.

[B9] Davis R. N. Nolen-Hoeksema S. (2000). Cognitive inflexibility among ruminators and nonruminators. *Cogn. Ther. Res.* 24 699–711.

[B10] Delis D. C. Kaplan E. Kramer J. H. (2001). *Delis-Kaplan Executive Function System (D-KEFS).* San Antonio, TX: Psychological Corporation.

[B11] Delis D. C. Kramer J. H. Kaplan E. Ober B. A. (2000). *CVLT-II: California Verbal Learning Test: Adult Version.* San Antonio, TX: Psychological Corporation.

[B12] Duff K. (2012). Evidence-based indicators of neuropsychological change in the individual patient: relevant concepts and methods. *Arch. Clin. Neuropsychol.* 27 248–261. 10.1093/arclin/acr120 22382384 PMC3499091

[B13] Elgamal S. Denburg S. Marriott M. MacQueen G. (2010). Clinical factors that predict cognitive function in patients with major depression. *Canad. J. Psychiatry* 55 653–661. 10.1177/070674371005501004 20964944

[B14] Groves S. J. Douglas K. M. Porter R. J. (2018). A systematic review of cognitive predictors of treatment outcome in major depression. *Front. in Psychiatry* 9:382. 10.3389/fpsyt.2018.00382 30210368 PMC6121150

[B15] Hagen B. I. Lau B. Joormann J. Småstuen M. C. Landrø N. I. Stubberud J. (2020). Goal Management Training as a cognitive remediation intervention in depression: a randomized controlled trial. *J. Affect. Disord.* 275 267–277. 10.1016/j.jad.2020.07.015 32734919

[B16] Hamilton K. E. Dobson K. S. (2002). Cognitive therapy of depression: pretreatment patient predictors of outcome. *Clin. Psychol. Rev.* 22 875–893. 10.1016/S0272-7358(02)00106-X 12214329

[B17] Hasselbalch B. J. Knorr U. Kessing L. V. (2011). Cognitive impairment in the remitted state of unipolar depressive disorder: a systematic review. *J. Affect. Disord.* 134 20–31. 10.1016/j.jad.2010.11.011 21163534

[B18] Huddy V. Reeder C. Kontis D. Wykes T. Stahl D. (2012). The effect of working alliance on adherence and outcome in cognitive remediation therapy. *J. Nerv. Ment. Dis.* 200 614–619. 10.1097/nmd.0b013e31825bfc31 22759940

[B19] Jacobson N. S. Truax P. (1991). Clinical significance: a statistical approach to defining meaningful change in psychotherapy research. *J. Consult. Clin. Psychol.* 59 12–19. 10.1037/0022-006x.59.1.12 2002127

[B20] Kabat-Zinn J. (1990). *Full Catastrophe Living: The Program of the Stress Reduction Clinic at the University of Massachusetts Medical Center.* New York, NY: Delacorte Press.

[B21] Kaser M. Zaman R. Sahakian B. J. (2017). Cognition as a treatment target in depression. *Psychol. Med.* 47 987–989. 10.1017/s0033291716003123 27938430

[B22] Koster E. H. W. Hoorelbeke K. Onraedt T. Owens M. Derakshan N. (2017). Cognitive control interventions for depression: a systematic review of findings from training studies. *Clin. Psychol. Rev.* 53 79–92. 10.1016/j.cpr.2017.02.002 28273486

[B23] Kurtz M. M. Seltzer J. C. Fujimoto M. Shagan D. S. Wexler B. E. (2009). Predictors of change in life skills in schizophrenia after cognitive remediation. *Schizophr. Res.* 107 267–274. 10.1016/j.schres.2008.10.014 19006657 PMC3399665

[B24] Levine B. Schweizer T. A. O’Connor C. Turner G. Gillingham S. Stuss D. T. (2011). Rehabilitation of executive functioning in patients with frontal lobe brain damage with goal management training. *Front. Hum. Neurosci.* 5:9. 10.3389/fnhum.2011.00009 21369362 PMC3043269

[B25] Lewandowski K. E. Sperry S. H. Cohen B. M. Norris L. A. Fitzmaurice G. M. Ongur D. (2017). Treatment to enhance cognition in bipolar disorder (TREC-BD): efficacy of a randomized controlled trial of cognitive remediation versus active control. *J. Clin. Psychiatry* 78 e1242–e1249. 29045770 10.4088/JCP.17m11476

[B26] Lindenmayer J. P. Fregenti S. Kang G. Ozog V. Ljuri I. Khan A. (2017). The relationship of cognitive improvement after cognitive remediation with social functioning in patients with schizophrenia and severe cognitive deficits. *Schizophr. Res.* 185 154–160. 10.1016/j.schres.2017.01.007 28094171

[B27] Listunova L. Bartolovic M. Kienzle J. Jaehn A. Grützner T. M. Wolf R. C. (2020). Predictors of cognitive remediation therapy improvement in (partially) remitted unipolar depression. *J. Affect. Disord.* 264 40–49. 10.1016/j.jad.2019.12.006 31846901

[B28] MacCallum R. C. Zhang S. Preacher K. J. Rucker D. D. (2002). On the practice of dichotomization of quantitative variables. *Psychol. Methods* 7 19–40. 10.1037/1082-989X.7.1.19 11928888

[B29] Marazziti D. Consoli G. Picchetti M. Carlini M. Faravelli L. (2010). Cognitive impairment in major depression. *Eur. J. Pharmacol.* 626 83–86. 19835870 10.1016/j.ejphar.2009.08.046

[B30] Medalia A. Richardson R. (2005). What predicts a good response to cognitive remediation interventions? *Schizophr. Bull.* 31 942–953. 10.1093/schbul/sbi045 16120830

[B31] Morimoto S. S. Wexler B. E. Liu J. Hu W. Seirup J. Alexopoulos G. S. (2014). Neuroplasticity-based computerized cognitive remediation for treatment-resistant geriatric depression. *Nat. Commun.* 5 1–7. 10.1038/ncomms5579 25093396 PMC4139707

[B32] Motter J. N. Pimontel M. A. Rindskopf D. Devanand D. P. Doraiswamy P. M. Sneed J. R. (2016). Computerized cognitive training and functional recovery in major depressive disorder: a meta-analysis. *J. Affect. Disord.* 189 184–191. 10.1016/j.jad.2015.09.022 26437233

[B33] Petersen J. Z. Porter R. J. Miskowiak K. W. (2019). Clinical characteristics associated with the discrepancy between subjective and objective cognitive impairment in depression. *J. Affect. Disord.* 246 763–774. 10.1016/j.jad.2018.12.105 30623822

[B34] Philippot P. Brutoux F. (2008). Induced rumination dampens executive processes in dysphoric young adults. *J. Behav. Ther. Exp. Psychiatry* 39 219–227. 10.1016/j.jbtep.2007.07.001 17698028

[B35] Porter R. J. Bourke C. Carter J. D. Douglas K. M. McIntosh V. V. W. Jordan J. (2016). No change in neuropsychological dysfunction or emotional processing during treatment of major depression with cognitive–behaviour therapy or schema therapy. *Psychol. Med.* 46 393–404. 10.1017/s0033291715001907 26446709

[B36] Pu S. Noda T. Setoyama S. Nakagome K. (2018). Empirical evidence for discrete neurocognitive subgroups in patients with non-psychotic major depressive disorder: clinical implications. *Psychol. Med.* 48 2717–2729. 10.1017/s003329171800034x 29679991

[B37] Quinn M. E. Keil D. C. Utke S. Joormann J. (2014). Trait rumination moderates the effect of executive control training. *J. Exp. Psychopathol.* 5 289–301. 10.5127/jep.038713

[B38] Reser M. P. Slikboer R. Rossell S. L. (2019). A systematic review of factors that influence the efficacy of cognitive remediation therapy in schizophrenia. *Austral. N. Z. J. Psychiatry* 53 624–641. 10.1177/0004867419853348 31177813

[B39] Rock P. L. Roiser J. P. Riedel W. J. Blackwell A. D. (2014). Cognitive impairment in depression: a systematic review and meta-analysis. *Psychol. Med.* 44 2029–2040. 10.1017/s0033291713002535 24168753

[B40] Roth R. M. Isquith P. K. Gioia G. (2005). *Behavioral Rating Inventory of Executive Function–Adult Version.* Lutz, FL: Psychological Assessment Resources.

[B41] Schulz K. F. Altman D. G. Moher D. (2010). CONSORT 2010 statement: updated guidelines for reporting parallel group randomised trials. *BMC Med.* 8:18. 10.1186/1741-7015-8-18 20334633 PMC2860339

[B42] Seccomandi B. Tsapekos D. Newbery K. Wykes T. Cella M. (2020). A systematic review of moderators of cognitive remediation response for people with schizophrenia. *Schizophr. Res.* 19:100160. 10.1016/j.scog.2019.100160 31828023 PMC6889639

[B43] Shilyansky C. Williams L. M. Gyurak A. Harris A. Usherwood T. Etkin A. (2016). Effect of antidepressant treatment on cognitive impairments associated with depression: a randomised longitudinal study. *Lancet Psychiatry* 3 425–435. 10.1016/s2215-0366(16)00012-2 26995298 PMC4860142

[B44] Siegle G. J. Ghinassi F. Thase M. E. (2007). Neurobehavioral therapies in the 21st century: summary of an emerging field and an extended example of cognitive control training for depression. *Cogn. Ther. Res.* 31 235–262. 10.1007/s10608-006-9118-6

[B45] Siegle G. J. Price R. B. Jones N. P. Ghinassi F. Painter T. Thase M. E. (2014). You gotta work at it: pupillary indices of task focus are prognostic for response to a neurocognitive intervention for rumination in depression. *Clin. Psychol. Sci.* 2 455–471. 10.1177/2167702614536160

[B46] Snyder H. R. (2012). Major depressive disorder is associated with broad impairments on neuropsychological measures of executive function: a meta-analysis and review. *Psychol. Bull.* 139 81–132. 10.1037/a0028727 22642228 PMC3436964

[B47] Stubberud J. Langenbahn D. Levine B. Stanghelle J. Schanke A.-K. (2013). Goal management training of executive functions in patients with spina bifida: a randomized controlled trial. *J. Int. Neuropsychol. Soc.* 19 672–685. 10.1017/s1355617713000209 23575309

[B48] Tornås S. Løvstad M. Solbakk A.-K. Evans J. Endestad T. Hol P. K. (2016). Rehabilitation of executive functions in patients with chronic acquired brain injury with goal management training, external cuing, and emotional regulation: a randomized controlled trial. *J. Int. Neuropsychol. Soc.* 22 436–452. 10.1017/s1355617715001344 26812574

[B49] Treynor W. Gonzalez R. Nolen-Hoeksema S. (2003). Rumination reconsidered: a psychometric analysis. *Cogn. Ther. Res.* 27 247–259.

[B50] Twamley E. W. Burton C. Z. Vella L. (2011). Compensatory cognitive training for psychosis: who benefits? Who stays in treatment? *Schizophr. Bull.* 37(Suppl._2), 55–62. 10.1093/schbul/sbr059 21860048 PMC3160125

[B51] Velligan D. I. Kern R. S. Gold J. M. (2006). Cognitive rehabilitation for schizophrenia and the putative role of motivation and expectancies. *Schizophr. Bull.* 32 474–485. 10.1093/schbul/sbj071 16641424 PMC2632243

[B52] Venezia R. G. Gorlyn M. Burke A. K. Oquendo M. A. Mann J. J. Keilp J. G. (2018). The impact of cognitive reserve on neurocognitive performance in major depressive disorder. *Psychiatry Res.* 270 211–218. 10.1016/j.psychres.2018.09.031 30267985

[B53] Vicent-Gil M. Keymer-Gausset A. Serra-Blasco M. Carceller-Sindreu M. de Diego-Adeliño J. Trujols J. (2018). Cognitive predictors of illness course at 12 months after first-episode of depression. *Eur. Neuropsychopharmacol.* 28 529–537. 10.1016/j.euroneuro.2018.02.001 29482974

[B54] Victor M. Lau B. Ruud T. (2016). Patient characteristics in a return to work programme for common mental disorders: a cross-sectional study. *BMC Public Health* 16:745. 10.1186/s12889-016-3431-0 27502950 PMC4977655

[B55] Vita A. Deste G. De Peri L. Barlati S. Poli R. Cesana B. M. (2013). Predictors of cognitive and functional improvement and normalization after cognitive remediation in patients with schizophrenia. *Schizophr. Res.* 150 51–57. 10.1016/j.schres.2013.08.011 23998953

[B56] Wechsler D. (1999). *Manual for the Wechsler abbreviated intelligence scale (WASI).* San Antonio, TX: The Psychological Corporation.

[B57] Wechsler D. (2014). *Wechsler adult intelligence scale–Fourth Edition (WAIS–IV).* San Antonio, TX: Psychological Corporation.

[B58] World Health Organization (2004). *ICD-10: International Statistical Classification of Diseases and Related Health Problems: Tenth Revision*, 2nd Edn Geneva: World Health Organization.

[B59] Wykes T. Huddy V. Cellard C. McGurk S. R. Czobor P. (2011). A meta-analysis of cognitive remediation for schizophrenia: methodology and effect sizes. *Am. J. Psychiatry* 168 472–485. 10.1176/appi.ajp.2010.10060855 21406461

